# Eye-movement indices of arousal predict ADHD and comorbid externalizing symptoms over a 2-year period

**DOI:** 10.1038/s41598-023-31697-3

**Published:** 2023-03-23

**Authors:** Johan Lundin Kleberg, Matilda A. Frick, Karin C. Brocki

**Affiliations:** 1 Department of Clinical Neuroscience, Centre for Psychiatry Research, Karolinska Institute, Gävlegatan 22, 113 33 Stockholm, Sweden; 2 Department of Psychology, Stockholm University, Stockholm, Sweden; 3 Department of Medical Sciences, Uppsala University, Uppsala, Sweden; 4 Department of Psychology, Uppsala University, Uppsala, Sweden

**Keywords:** Psychology, Preclinical research, Cognitive neuroscience, Diseases of the nervous system, Oculomotor system, Sensory processing

## Abstract

Attention-deficit/hyperactivity disorder (ADHD) follows a variable course across childhood. Disrupted arousal has been hypothesized to underlie core symptoms as well as comorbid internalizing and externalizing conditions. The current study examined eye-movement and pupil-dilation metrics indexing arousal as longitudinal predictors of ADHD, externalizing, and internalizing symptoms over a 2-year period. Participants aged 8–13 years (*N* = 54, 30% with a diagnosis of ADHD) completed a modified version of the gap-overlap task including arousal-inducing auditory warning signals. Parents rated symptoms at the time of testing and at 2 years follow-up. Phasic alerting (reaction-time reduction after alerting cues) is an index of arousal. Here, larger phasic alerting effects predicted higher ADHD-symptom levels 2 years later. Blunted pupil-dilation responses predicted externalizing symptoms at T2, controlling for ADHD and externalizing at T1. Our results support the theory that ADHD is associated with altered arousal. Blunted arousal reactivity may be a longitudinal risk factor for externalizing problems in children with ADHD symptoms.

## Introduction

The developmental trajectories of ADHD from childhood to adolescence are highly heterogeneous^1,2^ and remain poorly understood^1,3–6^ . About 1/3 of diagnosed children no longer fulfil diagnostic criteria by early adulthood^7^ . In this group, remission is particularly likely to occur in adolescence^8^ . The longitudinal development of ADHD is heterogeneous not only in terms of binary outcome (whether diagnostic criteria are fulfilled or not), but also in terms of symptom dimensions. A decrease in symptoms from childhood to adolescence is most likely to occur in the hyperactive/impulsive domain whereas levels of inattentive symptoms follow a more variable course^7,9^ . ADHD symptoms are continuously distributed in the general population^10^ . Higher symptom levels predict impairments across the symptom spectrum, with little evidence for a qualitative difference between individuals with a diagnosis and those with elevated symptom levels^11^ . Studies of symptom dimensions may therefore capture variation in symptomatology better than categorical differences between clinical and non-clinical groups.

The core symptoms of ADHD are inattention, hyperactivity, and impulsivity^12^ . Many, but not all, children with ADHD show signs of impaired attention and executive functions on neuropsychological tests^13^ .Temperamental patterns characterized by either increased negative affect and irritability or strong approach motivation and dysregulated positive affect known as exuberance are also common^6^ . Comorbid symptoms of internalizing disorders (such as generalized anxiety disorder, GAD) and externalizing disorders (especially oppositional defiant disorder, ODD) are highly prevalent, and as such likely to affect the longitudinal course of the disorder^14,15^ . For example, a recent study found that an increase in symptoms of GAD over a two-year period predicted worse outcome in terms of core ADHD symptoms^16^ . Internalizing symptoms are also a longitudinal predictor of cognitive impairment, beyond the influence of core ADHD symptoms^17^ . A better understanding of the factors that predict the longitudinal development of ADHD and comorbid symptoms during late childhood could potentially guide intervention and has therefore been identified as an important research priority^14^ .

Arousal refers to a set of interacting behavioral and neural systems^18^ . These are further divided into tonic and short-term (phasic) arousal^18^ . Behaviorally, short-term increases in phasic arousal enhances sensitivity to sensory information and response preparedness. Reduced baseline arousal and impaired arousal regulation have been reported in ADHD, although results are heterogeneous^19^ . Altered arousal may represent an underlying mechanism of core ADHD symptoms and associated cognitive impairments in areas such as sustained attention^14,20^ . As predicted by the dimensional perspective, individual differences in arousal were linked to concurrent variability in ADHD symptoms and cognitive impairments^21,22^ .

The view that arousal alterations underlie some of the attention impairments seen in ADHD are further supported by recent studies showing that at least some of these can be ameliorated by brief auditory warning signals, which increase arousal but do not provide information about the location or direction of visual targets^23,24^. This spatially non-informative warning signal effect is known as *phasic alerting*^25^ . The effect is believed to be modulated by short-term increases in cortical arousal and activity in neurotransmitter systems such as norepinephrine and dopamine^26,27^. Phasic alerting can therefore be seen as a modulation of attention by arousal induced through external sensory stimulation. In populations with reduced tonic arousal due to brain lesions^28^ or pharmacological manipulations^27^ , phasic alerting effects on attention tend to be increased. Phasic alerting could therefore be seen as a compensatory process, where externally triggered arousal responses compensate for a lack of endogenously generated phasic arousal.

In the current study, we tested whether phasic arousal alterations are not only concurrently, but also longitudinally linked to symptoms of ADHD and co-occurring internalizing and externalizing disorders over a two-year interval. Arousal was studied using two measures derived from eye tracking: warning signal modulation of gaze shifts (phasic alerting) and pupil dilation.

### Eye movements in ADHD

The fixated area of the visual field is highly prioritized for further cortical processing^29^, and eye movement alterations are therefore likely to bias information processing and learning. Since eye movements require very little or no verbal ability, they can be measured in individuals that vary widely in cognitive ability and age and are less vulnerable than self-report measures to memory biases and difficulties with introspection. The brain mechanisms underlying eye movements are partly overlapping with those involved in orienting of attention and are modulated by arousal^29,30^ . Therefore, eye movement studies may be informative about the attentional characteristics of ADHD and its underlying brain mechanisms^14,20,31,32^ .

Previous research has documented alterations in both voluntary generated and reflexive eye movements in children with either a diagnosis of ADHD or elevated ADHD symptoms, including increased gaze-shift reaction time^33–36^ , increased variability of saccadic latencies^37,38^, and difficulties to inhibit stimulus-driven gaze shifts^39,40^. Together, these eye-movement atypicalities point to possible alterations in brain networks involved in eye movement generation including a frontostriatal network involved in top-down control of eye movements and a widely distributed network including subcortical, cortical, and cerebellar regions involved in stimulus-driven eye movement generation^29^ .

### Pupil dilation

Pupil dilation is an index of arousal closely linked to activity in the locus coeruleus-noradrenergic (LC-NE) system^41^. LC-NE arousal amplifies neural reactivity to salient stimuli and dampens reactivity to non-relevant sensory input and is therefore a potential mechanism underlying the effects of arousal on attention^26,42,43^. Task-related phasic LC-NE arousal has a non-linear, inverted u-shaped relationship to tonic (baseline) activity^44^. That is, the largest phasic arousal responses are hypothesized to occur at intermediate levels of tonic arousal, whereas either too high or too low baseline arousal leads to small phasic responses.

A previous study in the current sample^23^ examined the latency of stimulus-driven gaze shifts in a group of 8–12-years old children (N = 71) of which 1/3 had a clinical diagnosis of ADHD. As expected, ADHD symptoms were associated with longer gaze-shift reaction times in the absence of warning cues. Auditory warning signals decreased gaze-shift reaction times in children with ADHD, and the strength of this effect was linearly related to the level of ADHD symptoms. Consistent with the theory that the effect of alerting cues was driven by changes in arousal, alerting cues induced strong pupil-dilation responses, an index of arousal modulated by LC-NE activity^45^ . However, at the individual level, no correlation was found between pupil-dilation amplitude and the reduction in gaze-shift reaction times after warning cues.

Pupil dilation is a relatively quick response sensitive to novelty and emotional valence^45^. Previous studies have indicated abnormal pupil dilation in ADHD, including increased reactivity to smiling faces^46^ and decreased tonic pupil size, indicating hypoarousal^47^ (for a review, see^19^). Wainstein and colleagues^47^ also found blunted phasic pupil dilation during a working memory task in individuals with ADHD of predominantly inattentive presentation compared to neurotypical controls. Pupil dilation was normalized by methylphenidate, which increases LC-NE and dopaminergic activity.

Research on the relation between arousal and ADHD symptoms is complicated by the fact that atypicalities within this domain have also been found in externalizing^48^ and internalizing^49^ disorders as well as other neurodevelopmental disorders such as autism^50^. Blunted autonomic nervous system (ANS) reactivity is seen in children with externalizing symptoms^48,51–53^, but it is debated whether this is best explained by the externalizing symptoms per se or by their overlap with ADHD^19^ . Increased autonomic reactivity and hyperarousal has been reported in internalizing disorders, although results are heterogeneous and differ between methods and tasks^42,49,52–54^ .

There are at least three potential roles that phasic alerting could play in the maintenance and developmental change of symptoms. First, it is possible that increased phasic alerting and symptoms have shared underlying mechanisms. In this case, increased phasic alerting should be associated with relatively higher symptom levels at follow-up. A second possibility is that phasic alerting can have a compensatory function for children with high levels of ADHD symptoms, and therefore predict a better longitudinal outcome of core or associated symptoms (see^1^ for a discussion about trait liability and compensatory mechanisms in ADHD). For example, children who are more responsive to phasic alerting cues may have a better ability to take advantage of external cues or sensory input to regulate their arousal, which could in turn predict a better functional outcome. A third possibility is that individual differences in phasic alerting do not predict ADHD symptoms per se but mediate the risk for comorbid externalizing or internalizing symptoms. Here, we tested these possibilities in a follow-up study of the sample reported in^23^ .

The following registered hypotheses were tested:

1. *Are eye movements predictive of ADHD symptoms at two-years follow up?* Based on the cross-sectional findings previously reported from the sample^23^ , we hypothesized that higher levels of ADHD symptoms at time point 2 (T2) would be predicted by (1) larger alerting effects, (2) relatively longer gaze-shift reaction times in the absence of warning cues, and (3) larger gaze-shift reaction-time variability at time point 1 (T1).
2. *Are eye movements predictive of change in ADHD symptoms?* We hypothesized that larger phasic alerting effects, longer gaze-shift reaction times, and higher gaze-shift reaction-time variability would also predict a smaller symptom reduction from T1 to T2.
3. *Is pupil dilation predictive of ADHD symptoms at 2-years follow up?* We hypothesized that the pupil dilation to alerting cues at T1 and would be linked to ADHD symptoms at T2. This hypothesis was left undirected since no relation between pupil dilation and symptoms of ADHD was found at T1^23^ .
4. *Is pupil dilation predictive of change in ADHD symptoms?* In an undirected analysis, we examined longitudinal links between pupil dilation to alerting cues at T1 and ADHD symptoms at T2.

*Are eye movements and pupil dilation predictive of comorbid symptoms and change in comorbid symptoms?* In exploratory (not registered) analyses, we examined whether eye-movement and pupil dilation metrics predicted longitudinal change in comorbid internalizing and externalizing symptoms, beyond effects attributable to ADHD symptoms.

## Methods

### Registered analysis plan

The analysis plan and hypotheses were registered on Open Science Framework prior to analyses (link: https://osf.io/9mh58/).

### Participants

The final sample included N = 54 with valid eye-tracking data and symptom measures from T1 and T2. The initial sample at T1 included N = 82 children (mean age 10.42 years; for a detailed description of the sample and recruitment process, see^23^). Of these, four were excluded because of lack of valid data, one because no symptom measures were available, and ten because they had taken stimulant medication (known to affect arousal) for ADHD at the day of testing. Families of thirteen children (five with ADHD) did not return the questionnaires or declined to participate at T2. The total sample at T2 therefore consisted of 54 children (16 with an ADHD diagnosis). Of these, three had missing data from the cued overlap condition, two from the uncued overlap condition, and one from the uncued gap condition, resulting in slightly different sample sizes in the various analyses. Demographic characteristics, symptom levels and the average number of valid trials are shown in Table 1. Participants with and without valid data at T2 did not differ in age at T1 or any of the symptom measures (all *p* > 0.14, see Supplement, S1).

**Table 1:** Demographic information, symptom ratings and number of valid trials.

	T1		T2		P
	Mean (SD)	Range	Mean (SD)	Range	
Demographics (T1)					
Age	10.55 (1.38)	8.00–13.00	12.53^+^ (1.45)	9.92–15^+^	< 0.001***
Sex (%female)	29%		29%	–	
IQ	9.75 (2.22)	3.5–14	–	–	
SES	4.29 (1.00)	2.25–5.50	–	–	
Symptoms					
ADHD	0.74 (0.72)	0.00–2.50	0.67 (0.62)	0.00–2.33	0.187
ADHD-I	0.79 (0.72)	0.00–2.67	0.79 (0.66)	0.00–2.44	0.974
ADHD-HI	0.69 (0.78)	0.00–2.78	0.55 (0.63)	0.00–2.33	0.020*
ODD	0.55 (0.55)	0.00–2.38	0.49 (0.53)	0.00–2.13	0.221
CD	0.05 (0.12)	0.00–0.60	0.06 (0.14)	0.00–0.60	0.529
GAD	1.64 (0.38)	1.17–2.50	1.60 (0.49)	1.00–3.17	0.320
Valid trials					
Overlap (silent)^a^ (max = 20)	12.46 (3.24)	5.00–18.00			
Overlap (cued)^b^ (max = 20)	13.68 (3.15)	5.00–18.00			
Gap^c^ (max = 20)	13.02 (3.27)	5.00–18.00			

*ADHD* attention-deficit/hyperactivity disorder, *ADHD-I* ADHD inattentive symptoms, *ADHD-HI* ADHD hyperactive/impulsive symptoms, *ODD* oppositional defiant disorder (measure of externalizing symptoms in the analyses), *CD* conduct disorder, *GAD* generalized anxiety disorder (measure of internalizing symptoms in the analyses).

*p < .05, ***p < .001.

^+^Refers to age at T2.

^a^*n* = 54.

^b^*n* = 53.

^c^*n* = 55.

Informed consent was given in written form by legal guardians of all participants. The study protocol was approved by the regional ethics review board (Etikprövningsnämnden) of Uppsala, Sweden and research was performed according to the relevant regulations and guidelines. Informed consent was obtained from participants and their legal guardians. The research was conducted in accordance with the Declaration of Helsinki.

### Symptom ratings and clinical characterization

Symptom measures were collected from parents at T1 and T2. Parents rated symptoms of ADHD using the ADHD Rating Scale-5 for Children and Adolescents^55^. Here, parents rate the degree to which their child shows each of the 18 ADHD symptoms listed in the Diagnostic and Statistical Manual for Mental Disorders, 5th Ed. (DSM-5; 9 each for the inattentive and hyperactive/impulsive symptom domains) on a four-graded Likert scale with scores for each item ranging from 0 to 3. Cronbach’s alpha for this measure was α = 0.95. Parental ratings of ODD using the Swanson, Nolan, and Pelham–IV (SNAP-IV) scale (eight items) were used as a measure of externalizing symptoms^56^ . ODD is the most common comorbid externalizing disorder in ADHD and is highly correlated with other symptom dimensions such as conduct disorder. Cronbach’s alpha was α = 0.93. Since symptoms of GAD are highly correlated with a common internalizing symptoms factor^57^ , the six-item generalized anxiety subscale of Spence Children’s Anxiety Scale (SCAS;^58^ ) was used as a measure of internalizing symptoms. Cronbach’s alpha was α = 0.77 at T1 and α = 0.86 at T2. Internalizing and externalizing symptoms were moderately correlated at T2 (r = 0.34, p = 0.007) whereas no significant correlation was seen at T1 (r = 0.25, p = 0.06).

All clinical data are expressed as mean scores and described in Table 1. As can be seen, ADHD symptom levels did not decrease significantly from T1 to T2. Separate analyses of the inattentive and hyperactive/impulsive symptom domains showed significant reductions in the latter but not the former, replicating previous studies^14,59^. There was a near-significant reduction in ODD symptoms. Parents of children with ADHD (n = 16) confirmed that their children had received a diagnosis of ADHD in regular care by a psychologist or physician. At T1, nine participants were treated with stimulant or non-stimulant medication and at T2, five children were on medication according to parent report. Two children had received other forms of treatment by a psychologist (n = 1) or counsellor (n = 1), and one child was treated for Hashimoto’s disease. Two participants with ADHD had a comorbid autism spectrum disorder.

### Experimental task

**Figure 1:**
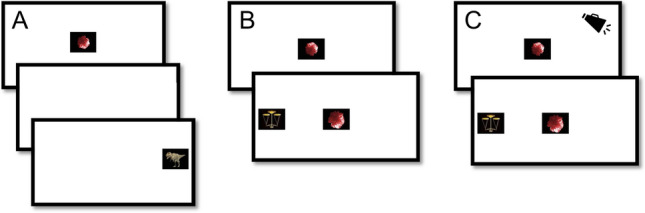
Overview of the eye-tracking task. The central stimulus was presented during a variable interval ranging from 800 to 1700 ms. In the gap condition (**A**), the central stimulus was extinguished before the onset of the peripheral stimulus during a period ranging from 120 to 200 ms. In the uncued overlap condition (**B**), the central stimulus remained on screen after the onset of the peripheral stimulus. In the cued overlap condition (**C**), auditory alerting cues were presented at variable time intervals ranging between 500 and 0 ms before the onset of the peripheral stimulus on 50% of the overlap trials. Participants completed 60 trials (20 gap, 20 uncued overlap, 20 cued gap).

The experimental task was an adapted version of the gap-overlap task (see Fig. 1). In this task, participants initially fixate a stimulus in the center of a screen, and then initiate a gaze shift to an upcoming peripheral stimulus. Gaze-shift reaction times are measured under two conditions: gap trials, where the central target is extinguished before the onset of the target (creating a temporal “gap”) and overlap trials, where the central stimulus remains on the screen during the onset of the target. Reaction times are typically longer during overlap trials, an effect which results from a combination of two phenomena. First, the focus of visual attention must be disengaged from the central stimulus during overlap trials, which is associated with a time cost. Secondly, the disappearance of the central stimulus during gap trials functions as a spatially non-specific warning cue which increases wakefulness and arousal and decreases reaction time. The gap effect is considerably decreased by alerting cues presented shortly before the onset of the peripheral stimulus in the overlap condition. The warning signal component of the gap effect can therefore be examined by comparisons between cued and non-cued overlap trials. Trials from the three conditions were presented in pseudorandom order in a single block.

### Recording and processing of eye-tracking data

Eye-tracking data were recorded with a corneal-reflection eye tracker (Tobii T120, Tobii Inc, Danderyd, Sweden) at a sample rate of 60 HZ. Testing took place in a university lab facility or in an outpatient pediatric clinic. Data were processed as described in a previous study reporting results from this sample at T1^23^. Gaze shifts were identified using an I-VT filter algorithm with the following steps. First, gaze velocity was smoothed using a moving average filter covering 50 ms. Subsequently, the start of a gaze shift was detected when gaze velocity exceeded 30°/s. Gaze shift latency was defined as the time from the onset of the peripheral stimulus to the time when the point of gaze first left the central stimulus (see Fig. 1). Trials were discarded if an anticipatory gaze shift defined as latencies shorter than 120 ms were detected. Following visualizations of the data, we also discarded a small number of unlikely long gaze shift (> 874 ms, or the 99th percentile).

Pupil data were processed using the following steps. First, gaps shorter than 100 ms were covered by linear interpolation. Secondly, pupil-size was filtered using a moving median filter with a window size corresponding to 80 ms (see Supplement, S2). Pupil-dilation amplitude was defined as the median size of the pupil during a 0–1500 ms time window after the onset of the visual stimuli, baseline corrected to the median pupil size during a 333 ms (20 samples) baseline period. Following recent recommendations^60^, baseline correction was conducted through subtraction. The baseline period was defined as the 1000–666 ms interval before stimulus onset, i.e., before the onset of the alerting cues. Trials with < 50% valid samples were discarded.

The *alerting effect* was defined as the difference in mean gaze shift reaction time between the uncued and the cued overlap conditions, with higher values indicating a larger reduction in reaction times following alerting cues. *Reaction-time variability* was defined as the interquartile range of all gaze shift latencies in the absence of alerting cues, fitted for each individual.

We aimed to keep ambient illuminance constant at around 500 lx. This was not possible in all cases due to room changes. Of the final data set, 5 participants were tested at ambient illuminance < 450 lx and 7 > 550 lx. No relation between ambient lux level and pupil-dilation response was found (*r* = −0.21, *p* = 0.14).

### Statistical analyses

The main analyses were conducted in two steps. First, we tested whether the eye tracking metrics predicted symptom levels at T2 using linear regressions. In a second step, we tested whether the experimental variables identified as significant predictors of symptoms at T2 also predicted the degree of change in symptoms from T1 to T2. These analyses were conducted by adding symptoms at T1 and the experimental variable of interest as simultaneous predictors in a regression model with symptoms at T2 as outcome variable, following previous recommendations^61^ . In other words, these analyses test whether an experimental measure explains variance in symptoms at T2 over and beyond what is explained by symptoms at T1.

All experimental variables were screened for outliers. One participant with a pupil-dilation response > 3.7 standard deviations above the mean was excluded from analyses of this measure. After this, no variable deviated significantly from the normal distribution (Shapiro–Wilk test, all p > 0.06). Violin plots showing the distribution of the experimental variables are shown in Supplement, S3.

Statistical analyses were conducted in R^62^ . Analyses testing relations between experimental variables and symptom measures were conducted after averaging data at the level of the individual. Outlier observations defined as standardized residuals ± 3.25 were removed (see Tables 2, 3 and 4 for the number of removed observations). Residual plots of all statistical models indicated that the data conformed to the assumptions of normality. However, because gaze-shift reaction times often deviate from a normal distribution, we validated the analyses by repeating all analyses using individual medians rather than means as measures of central tendency. This did not change any of the results. In an additional validation analysis, data were reanalyzed using general linear models with a log link (see Supplementary materials, S4). This did not change the significance of any of the results. The median difference in Akaike information criterion (AIC) values between models fitted with a log and identity link was 0.03, indicating very small differences in model fit. Additional validation analyses (see Supplement, S5) were conducted to examine change in pupil dilation and gaze shift reaction times over the course of the experiment. These analyses showed no change in gaze-shift reaction times during the task but a decrease in pupil-dilation responses to alerting cues. However, no interaction effects between trial and ADHD were found, indicating that the degree of change in the experimental variables did not vary with symptom level. Based on previously reported data from T1, we did not hypothesize links between gaze-shift latencies in the cued overlap condition and the examined symptom dimensions. Analyses related to this measure are therefore reported as supplementary materials (Supplement, S6).

Effect sizes are reported as standardized *β* coefficients. A power analysis using the R library *pwr* showed that the study had 80% power to detect medium effect sizes of β = 0.37 or above. The significance level was set to 0.05. P-values were corrected for multiple comparisons using the Bonferroni procedure for each symptom measure and analysis (i.e., five comparisons).

## Results

*Are eye movements predictive of ADHD symptoms at 2-years follow up?* Larger alerting effects predicted higher symptom levels of ADHD at T2 [β = 0.48, *t* (46)  = 3.68, *p* = 0.003]. ADHD symptoms at T2 were also predicted by gaze-shift reaction times at T1 in the uncued overlap condition [β = 0.56, t (46) = 4.63, *p* < 0.001], but not in the uncued gap condition [β = 0.27, *t* (48) = 1.95, *p* = 0.283]. Gaze shift reaction-time variability in the uncued conditions was also linked to ADHD at T2 [β = 0.51, *t* (48) = 4.07, *p* = 0.001].

*Are eye movements predictive of change in ADHD symptoms from T1 to 2-years follow up?* Change in ADHD symptoms was uniquely predicted by longer gaze-shift latency in the uncued overlap condition [β = 0.55, *t* (45) = 2.69, *p* = 0.050] and by higher gaze shift variability condition [β = 0.66, *t* (47) = 2.98, *p* = 0.023] after controlling for the same symptom dimension at T1. No other relations between eye movements and change in ADHD symptoms were found (see Table 2).

**Table 2:** Relations between eye-movement measures, symptoms of ADHD at T2, and change in ADHD symptoms from T1 to T2.

	b	β	t	df	P (Bonferroni)
Relation to ADHD symptoms at T2					
Alerting effect	58.01	0.48	3.68	46	0.003**
Gaze-shift latency (uncued overlap)^‡^	65.27	0.56	4.63	46	< 0.001***
Gaze-shift latency (uncued gap)	17.58	0.27	1.95	48	0.283
Gaze-shift variability (uncued)	55.23	0.51	4.07	48	0.001**
Pupil-dilation response (cued overlap)	−0.02	−0.14	−0.96	47	> 0.900
Relation to ADHD change					
Alerting effect	46.97	0.39	1.73	45	0.452
Gaze-shift latency (uncued overlap) ‡	64.25	0.55	2.69	45	0.050*
Gaze-shift latency (uncued gap)	22.78	0.35	1.42	47	0.806
Gaze-shift variability (uncued)	72.49	0.66	2.98	47	0.023
Pupil-dilation response (cued overlap)	0	−0.04	−0.14	46	> 0.90

*ADHD* attention-deficit/hyperactivity disorder.

*p < 0.05, **p < 0.01, ***p < 0*.*001 (Bonferroni-corrected).

^‡^1 outlier observation removed. Unstandardized *b* coefficients indicate the predicted change in ms (gaze-shift latency) or pupil size (mm).

*Is pupil dilation predictive of ADHD symptoms at 2-years follow up?* No relation was found between pupil dilation at T1 and ADHD symptoms at T2, [β = −0.14, *t* (47) = 0.96, *p* > 0.900].

*Is pupil dilation predictive of change in ADHD symptoms from T1 to 2-years follow up?* Pupil dilation at T1 did not predict ADHD symptoms at T2 after controlling for ADHD symptoms at T1, [β = −0.04, *t* (46) = 0.14, *p* > 0.900].

*Are eye movements predictive of comorbid symptoms at 2-years follow up?* Eye movements at T1 did not predict internalizing or externalizing symptoms at T2 (all p-values > 0.900 after Bonferroni correction, see Tables 3 and 4).

**Table 3:** Relations between eye-movement measures, externalizing symptoms (EXT) at T2 and change in EXT symptoms from T1 to T2.

	b	β	t	df	P (Bonferroni)
Relation to EXT symptoms at T2					
Alerting effect	9.98	0.08	0.43	45	> 0.90
Gaze-shift latency (uncued overlap)^‡^	15.03	0.12	0.68	45	> 0.90
Gaze-shift latency (uncued gap)	−0.64	−0.01	−0.04	47	> 0.90
Gaze-shift variability (uncued)	−11.72	−0.1	−0.52	47	> 0.90
Pupil-dilation response (cued overlap)	−0.08	−0.58	−2.94	46	0.026*
Relation to EXT change					
Alerting effect	9.54	0.07	0.29	43	> 0.90
Gaze-shift latency (uncued overlap)^‡^	−13.54	−0.11	−0.44	43	> 0.90
Gaze-shift latency (uncued gap)	2.53	0.04	0.12	45	> 0.90
Gaze-shift variability (uncued)	8.07	0.07	0.26	45	> 0.90
Pupil-dilation response (cued overlap)	−0.14	−1	−3.72	44	0.003**

*EXT* externalizing symptoms.

*p < 0.05 (Bonferroni-corrected).

^‡^1 outlier observation removed. Unstandardized *b* coefficients indicate the predicted change in ms (gaze shift latency) or pupil size (mm).

**Table 4:** Relations between eye-movement measures, internalizing symptoms (INT) at T2 and change in INT symptoms from T1 to T2.

	b	β	t	df	P (Bonferroni)
Relation to INT symptoms at T2					
Alerting effect	−27.75	−0.21	−1.43	45	0.799
Gaze-shift reaction time (silent overlap)‡	−5.57	−0.04	−0.31	45	> 0.90
Gaze-shift reaction time (silent gap)	−17.95	−0.23	−1.5	47	0.699
Gaze-shift variability (uncued)	0.09	0	0	47	> 0.90
Pupil-dilation response	0.01	0.06	0.34	46	0.366
Relation to INT change					
Alerting effect	−61.51	−0.47	−2.44	43	0.095
Gaze-shift latency (silent overlap)	−28.37	−0.22	−1.15	43	> 0.90
Gaze-shift latency (gap)	−0.84	−0.01	−0.05	45	> 0.90
Gaze-shift variability (uncued)	5.71	0.04	0.22	45	> 0.90
Pupil-dilation response (cued overlap)	−0.01	−0.08	−0.34	44	> 0.90

*INT* internalizing symptoms.

*p < 0.05 (Bonferroni-corrected).

^‡^1 outlier observation removed. Unstandardized *b* coefficients indicate the predicted change in MS (gaze shift latency) or pupil size (mm).

*Are eye movements predictive of change in comorbid symptoms from T1 to 2-years follow up?* After Bonferroni corrections, eye movements at T1 did not predict internalizing or externalizing symptoms at T2 after controlling for the same symptom dimension at T1 (all p-values > 0.090, see Tables 3 and 4).

*Is pupil dilation predictive of comorbid symptoms at 2-years follow up?* Smaller pupil-dilation responses at T1 predicted higher externalizing symptom levels at T2, after controlling for ADHD symptoms at T2 [β = −0.58, *t* (46) = −2.94, *p* = 0.026]. No relation was found between pupil dilation at T1 and internalizing symptoms at T2, controlling for ADHD symptoms at T2 [β = 0.06, *t* (46) = 0.34, *p* > 0.900].

*Is pupil dilation predictive of change in comorbid symptoms from T1 to 2-years follow up?* Smaller pupil-dilation responses at T1 predicted higher externalizing symptom levels at T2, after controlling for externalizing symptoms at T1 and ADHD symptoms at T1 and T2 [β = −1.00, *t* (44) = −3.72, *p* = 0.003, see Table 3]. No relationship was found between pupil-dilation responses at T1 and change in internalizing symptoms at T2 [β = −0.08, *t* (44) = −0.34, *p* > 0.900, see Table 3].

## Discussion

Disrupted arousal is hypothesized to be a mechanism underlying ADHD symptoms and related visual attention and eye-movement alterations. Disrupted arousal may also underlie associated symptoms of externalizing and internalizing disorders. In this study, we examined whether individual differences in eye-movement metrics sensitive to arousal predict change in ADHD symptoms and comorbid internalizing and externalizing disorders over a 2-year period in a group of children oversampled for a diagnosis of ADHD. The *phasic alerting effect* is defined as a decrease in response time after warning signals, caused by a temporary increase in arousal. The magnitude of this effect was previously found to be associated with higher levels of ADHD symptoms)^23^. In the current study, we show that this measure also predicts symptom levels two years later. However, phasic alerting effects did not predict the degree of change between these time points, suggesting that altered arousal is a trait-like vulnerability factor for ADHD symptoms during late childhood and early adolescence rather than a mechanism of change. Our results therefore support the theory that arousal alterations underlie symptoms of ADHD^14,20^.

Contrary to our expectations, the pupil-dilation response to auditory cues was not linked to ADHD symptoms, but instead predicted externalizing symptoms at T2 beyond what could be explained by core ADHD symptoms. Pupil-dilation responses at T2 were also linked to the degree of change in externalizing symptoms from T1 to T2, so that children with lower pupil-dilation responses were more likely to have higher levels of externalizing symptoms at T2, even after controlling for the same symptom dimension at T1 as well as for ADHD symptoms at T1 and T2. To our knowledge, this is the first study to examine longitudinal links between pupil dilation in mid childhood and subsequent externalizing symptoms. Pupil dilation is modulated by activity in the cholinergic and noradrenergic systems^41^. Tentatively, our results therefore suggest alterations in these systems that are relatively specific for externalizing disorders as compared to ADHD symptoms.

### Implications for arousal hypotheses of ADHD

Studies in typically developing populations show that phasic alerting can improve attention and perception^63,64^, and thereby affect learning. Phasic alerting effects could therefore potentially have a compensatory role for children with ADHD. Consistent with this theory, stimulant medication for ADHD, such as methylphenidate (MPH) is known to increase arousal and has dose-dependent beneficial effects on several neurocognitive functions closely linked to arousal, such as vigilance and response-time variability in children with ADHD^65^. Sustained periods of background white noise can be beneficial for cognitive performance in children with ADHD, possibly through increased arousal^24^. Our results indicate that beneficial effects of increased arousal on visual attention in children with high levels of ADHD symptoms are seen already during a very short time scale. An interesting question for future studies is to examine whether phasic alerting either predicts response to medication and other treatments or changes after treatment.

Stronger alerting effects (greater reduction of gaze-shift reaction times after warning signals) predicted higher ADHD symptom levels at 2-years follow up. Phasic alerting can have beneficial effects on perception and learning^26,63,64^, and could therefore potentially compensate for risk factors for development of internalizing symptoms associated with ADHD, such as peer and learning problems.

### Is low arousal a risk factor for continuing externalizing symptoms?

Externalizing symptoms tend to decrease from childhood to adolescence, but the longitudinal course is variable and a subset of children with ADHD show a progressive course^14^. Here, reduced pupil-dilation responses to auditory cues predicted a relative increase in externalizing symptoms over two years, after control for ADHD and externalizing symptoms at T1. This suggests that blunted pupillary reactivity may contribute to the maintenance or increase of externalizing symptoms during mid childhood. As noted in the introduction, previous studies have reported reduced arousal reactivity to negative emotional stimuli or unexpected sensory information as measured by electrodermal activity, electrocardiography and electroencephalogram (EEG) in children with conduct problems^48,52^ . However, the symptomatic specificity and longitudinal links of these measures remains debated^19,48^ . Our results also suggest that the link between blunted arousal reactivity and externalizing symptoms extends beyond emotional stimuli. Pupil dilation is elicited by novelty and unexpected stimuli and is closely linked to activity in the brains noradrenergic system^26,45^ .

As described in the introduction, the phasic alerting effect is defined as the difference between gaze-shift latency with and without warning cues. The latter of these components—gaze-shift reaction times without warning signals—has consistently been linked to concurrent ADHD symptoms^33^. As predicted, longer gaze-shift reaction times in the absence of warning signals predicted ADHD symptoms at 2-years follow up. This link was found when gaze had to be disengaged from a previously fixated location (i.e., in the uncued overlap condition), but not as predicted in the gap condition (where the initially fixated central stimulus is removed prior to the gaze shift). This suggests that impaired disengagement of visual attention rather than slow gaze-shift reaction time per se may a risk-factor for continuing ADHD symptoms. This result is consistent with previous reports of reduced gaze-shift reaction times in ADHD. Previous studies have shown that ADHD symptoms are associated with increased gaze-shift reaction time variability^23,37,38^. Here, we found that this measure also predicted the longitudinal development of ADHD, so that children with increased response-time variability at T1 had higher ADHD symptoms at T2, even after controlling for T1 symptoms.

### Limitations

Some limitations should be mentioned. First, the sample size was relatively small. The naturalistic study design means that the type of treatments received between T1 and T2 was not controlled for. The study did not include a measure of tonic arousal, which could have provided valuable information about potential interactions between tonic and phasic arousal. Due to the relatively low sample rate, we were not able to analyze potentially informatively eye-movement metrics such as microsaccade rate or saccadic velocity. Importantly, all significant effects were considerably larger than the sampling error (i.e., the average expected difference between a recorded and an actual event, 8.33 ms), meaning that temporal imprecision in the equipment is unlikely to have influenced the results. Inclusion of cued gap trials may have provided a more complete picture of the effects of alerting on gaze shift reaction times. It should also be noted that, since the current study examined the longitudinal course of ADHD symptoms across the whole spectrum, more research is needed to determine whether the findings also hold for classification of remission versus persistence of categorical diagnoses in clinical settings. Findings from the current study point to a potential mechanism underlying externalizing disorders, which is not shared with ADHD or internalizing symptoms. However, it should be noted that both externalizing and internalizing symptoms were measured using relatively brief instruments. Studies using broader measures of internalizing and externalizing symptoms may therefore give a more complete picture of their relationship with altered arousal processes.

## Supplementary Information

Supplementary Information.

## Data Availability

The datasets generated during and/or analyzed during the current study are not publicly available due to restrictions in the ethical permit but are available from the corresponding author on reasonable request.
