# Supplementary material for: Eye-movement indices of arousal predict ADHD and comorbid externalizing symptoms over a 2-year period
Source: Sci Rep. 2023 Mar 23;13:4767. doi: 10.1038/s41598-023-31697-3 (PMC10036637; doi:10.1038/s41598-023-31697-3)
Supplement: Supplementary file 1 — Supplementary Information. [file 41598_2023_31697_MOESM1_ESM.docx]

Supplementary Materials

S1. Comparison between participants with and without valid data at T2

Of the participants with valid eye tracking data at T1, 13 participants did not complete the assessment at T2. As can be seen in *Table S1,* these participants did not differ significantly from the participants with valid data at T2 in age or any of the symptom measures at T1.

| Variable (T1) | Assessment at T2 completed  M (sd) | Assessment at T2 not completed  M (sd) | t | p |
| --- | --- | --- | --- | --- |
| Age | 10.55 (1.36) | 10.12 (1.5) | 0.96 | .349 |
| ADHD | 0.74 (0.71) | 0.99 (0.74) | -1.07 | .298 |
| ADHD-HI | 0.69 (0.77) | 0.77 (0.66) | -0.41 | .684 |
| ADHD-I | 0.79 (0.71) | 1.2 (0.87) | -1.56 | .139 |
| GAD | 1.65 (0.39) | 1.56 (0.56) | 0.51 | .614 |
| ODD | 0.55 (0.54) | 0.86 (0.69) | -1.54 | .144 |

***Table S1.*** *Descriptive statistics and statistical comparisons between participants included in the analysis and participants with valid data at T1 who did not complete the assessment at T2. ADHD = Attention deficit/hyperactivity disorder; ADHD-I = ADHD inattentive symptoms; ADHD-HI = ADHD hyperactive/impulsive symptoms; ODD = oppositional defiant disorder (Measure of externalizing symptoms in the analyses); CD = conduct disorder; GAD = Generalized anxiety disorder (Measure of internalizing symptoms in the analyses)*

S2. Pupil filtering

*Figure S1* shows the effect of the applied filter on the pupil signal for a sample participant. As can be seen, the filter produces a smoother signal and reduces short-term variability.

**
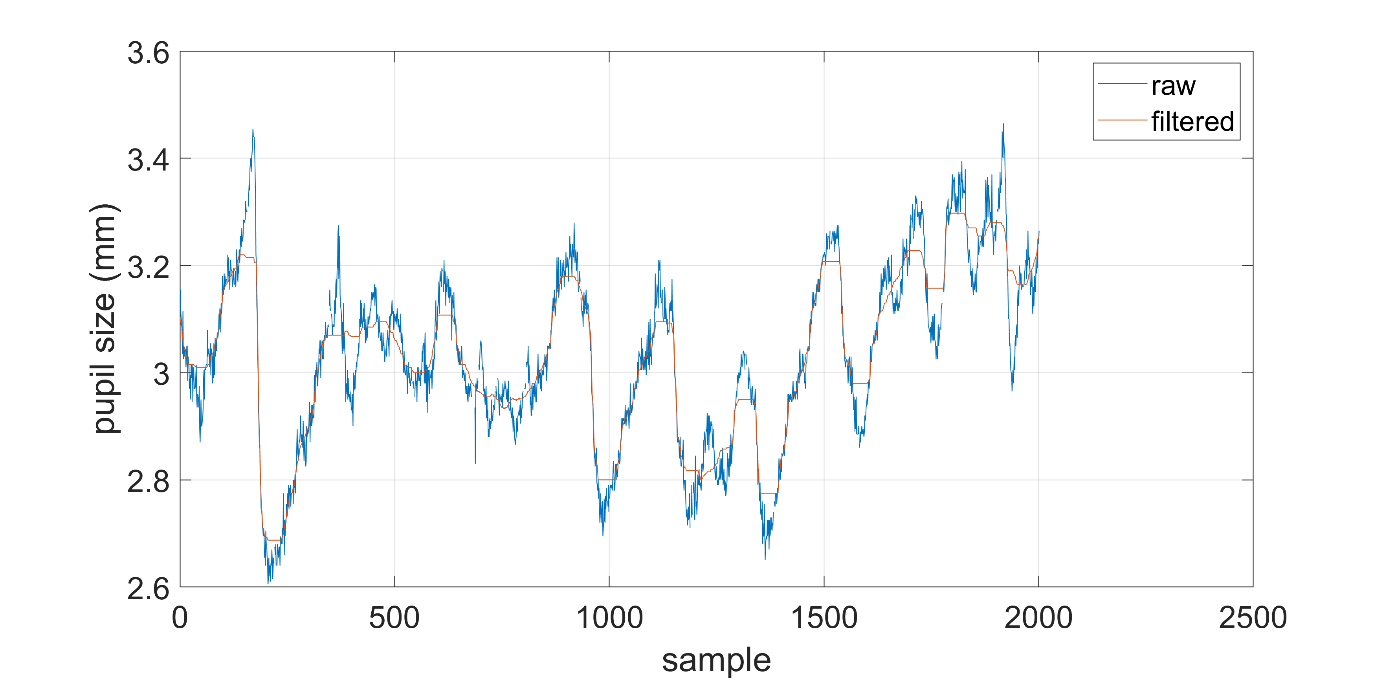
**

*Figure S1. Raw and filtered pupil trace during 2000 samples for one participant*

S3. Visualizations of data distributions

**
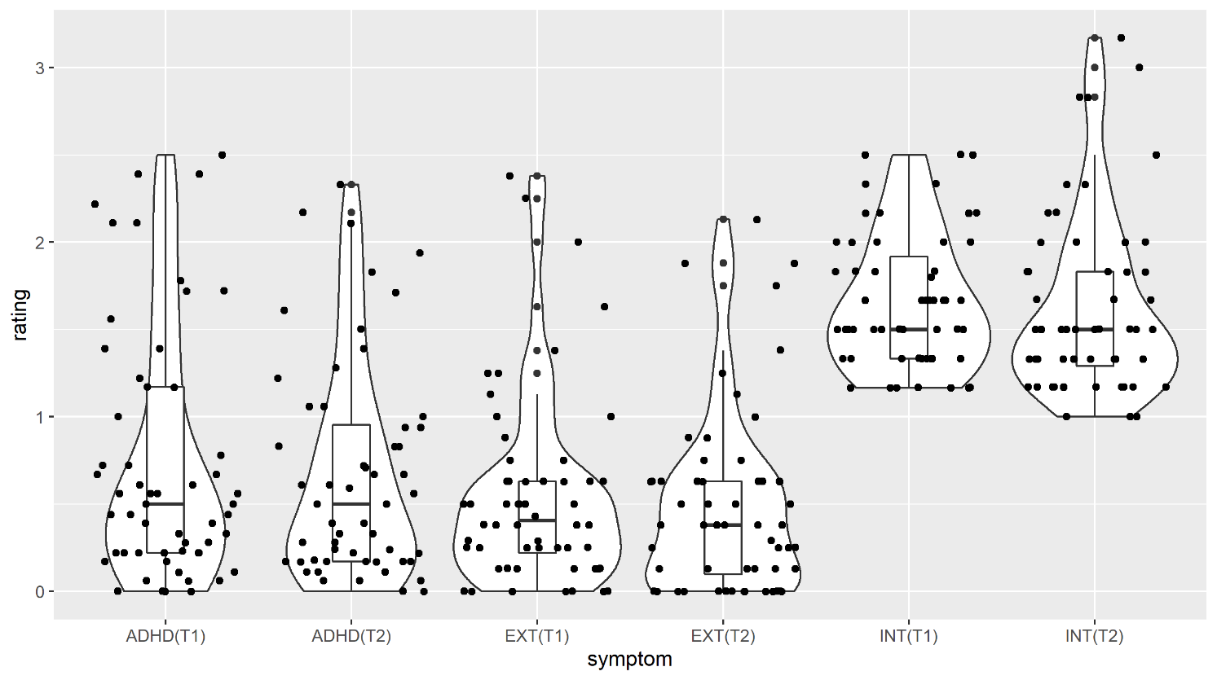
**

*Figure S2.* Symptom ratings at T1 and T2. The dots show individual data points. ADHD = Attention deficit/hyperactivity disorder; EXT = Externalizing symptoms; INT = Internalizing symptoms. T1 = Timepoint 1; T2 = Timepoint 2

**
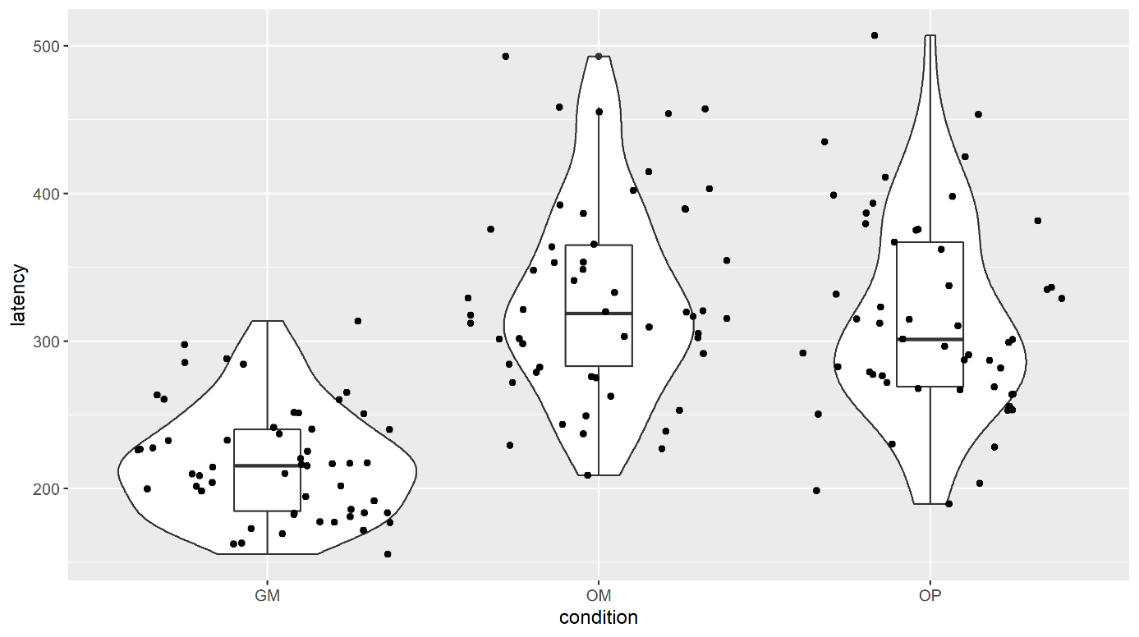
**

*Figure S3. Gaze shift latency by condition. Dots show individual data points. GM = gap, OM = uncued overlap, OP = cued overlap.*

**
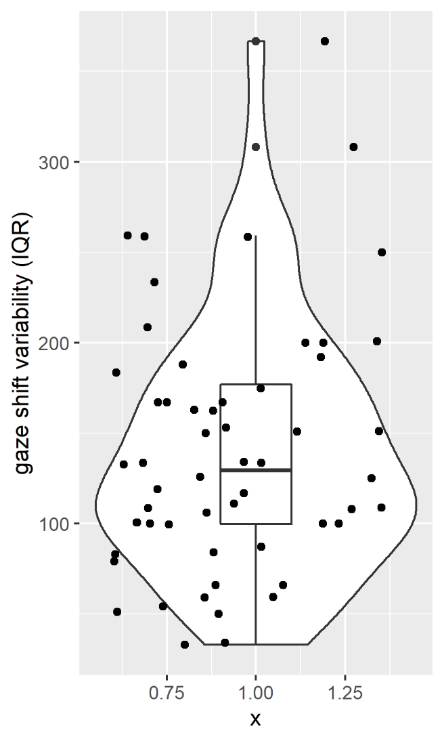
**

*Figure S4. Gaze shift variability (Inter-quartile range in milliseconds). Dots show individual participants.*

**
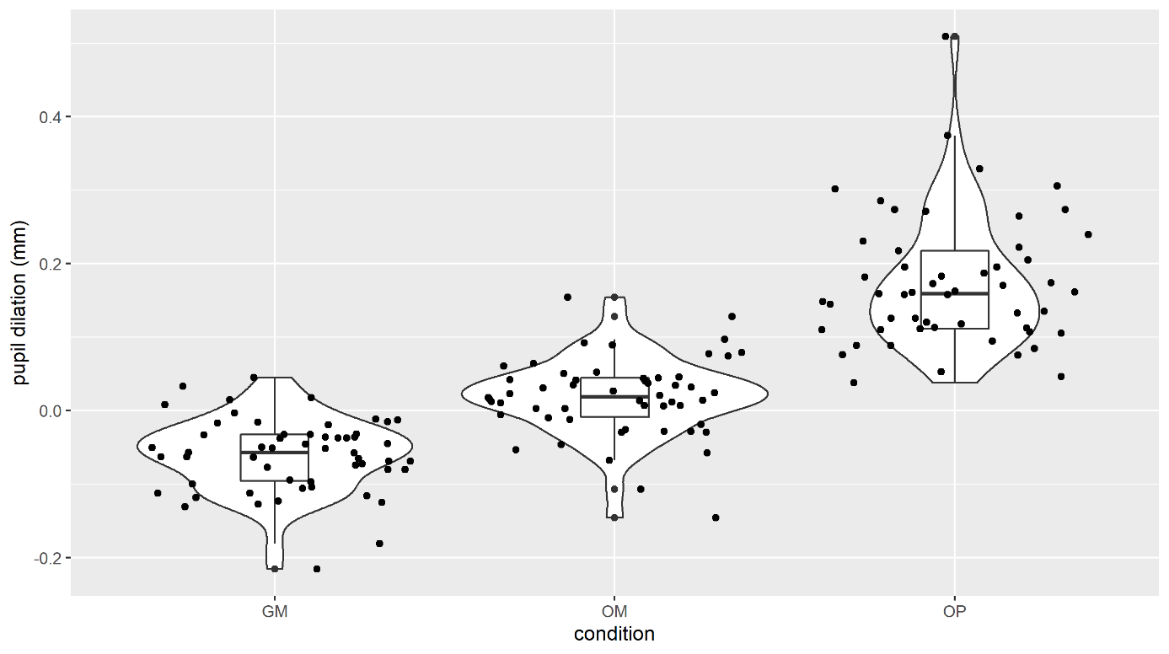
**

*Figure S5 Pupil dilation (mm) by condition. Dots show individual participants.*

S4. Alerting effect and pupil dilation in excluded participants taking stimulant medication at the day of testing

Five individuals had taken stimulant medication at the day of testing at T1 and had completed the follow-up assessment at T2. These participants were excluded from the analysis, since stimulant medication is known to affect arousal. Descriptive statistics for these participants are shown in *Figure S6.* Due to the small sample size, no inferential statistical tests were conducted.

*
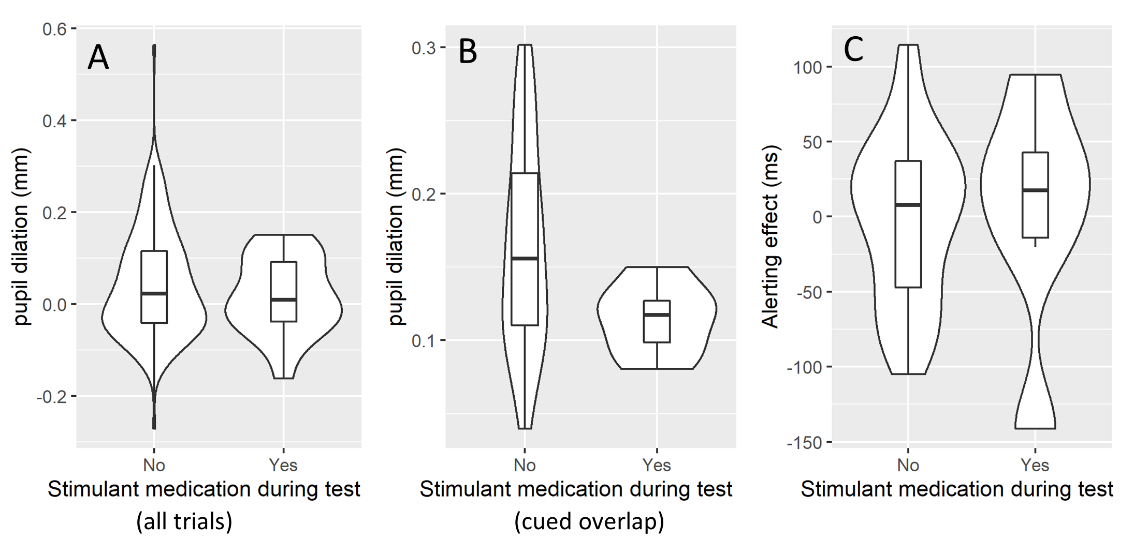
*

***Figure S6****. Pupil dilation responses during all trials (A), cued overlap trials (B), and alerting effects (C) in participants with valid clinical data at T2 who had not taken stimulant medication at the day of testing (n = 54) and excluded participants on stimulant medication at the day of testing (n = 5).*

S5. Reanalysis of the data using general linear models (GLMs) fitted with a ‘log’ link

*Table S2. Relations between eye movement measures, symptoms of ADHD at T2, and change in ADHD symptoms from T1 to T2 (General linear models fit with* ***log link*)**

|  | **b** | **β** | **t** | **df** | **P (Bonferroni)** | **ΔAIC (identity – log link)** |
| --- | --- | --- | --- | --- | --- | --- |
| **Relation to ADHD symptoms at T2** |  |  |  |  |  |  |
| Alerting effect | 0.06 | 0 | 3.82 | 46 | **.002**** | 0.23 |
| Gaze shift latency (uncued overlap) ‡ | 0.1954 | 0 | 4.86 | 46 | **<.001***** | -0.19 |
| Gaze shift latency (uncued gap) | 0.09 | 0 | 2 | 48 | .257 | -0.03 |
| Gaze shift variability (uncued) | 0.33 | 0 | 4.21 | 48 | **.001**** | -0.84 |
| Pupil-dilation response (cued overlap) | -0.11 | -0.85 | -0.92 | 47 | >.90 | 0.00 |
|  |  |  |  |  |  |  |
| **Relation to ADHD change** |  |  |  |  |  |  |
| Alerting effect | 0.05 | 0 | 1.8 | 45 | .395 | 0.24 |
| Gaze shift latency (uncued overlap) ‡ | 0.18 | 0 | 2.75 | 45 | **.043*** | -0.14 |
| Gaze shift latency(uncued gap) | 0.11 | 0 | 1.51 | 47 | .687 | 0.02 |
| Gaze shift variability (uncued) | 0.44 | 0 | 3.42 | 47 | **.006**** | -0.63 |
| Pupil-dilation response (cued overlap) | -0.04 | -0.27 | -0.17 | 46 | >.90 | 0.00 |

* *p* < .05, ** *p* <.01, *** *p <.*001 (Bonferroni-corrected). ADHD = Attention deficit/hyperactivity disorder. ‡ 1 outlier observation removed. Unstandardized *b* coefficients indicate the predicted change in ms (gaze shift latency) or pupil size (mm). ΔAIC = difference in Aikaike information criterion (AIC) between GLMs fitted with identity versus log link.

*Table S3. Relations between eye movement measures, externalizing symptoms (EXT) at T2, and change in EXT symptoms from T1 to T2. (General linear models fit with* ***log link*)**

|  | **b** | **β** | **t** | **df** | **P (Bonferroni)** | **ΔAIC (identity – log link)** |
| --- | --- | --- | --- | --- | --- | --- |
| **Relation to EXT symptoms at T2** |  |  |  |  |  |  |
| Alerting effect | 0.01 | 0 | 0.39 | 45 | >.90 | 0.20 |
| Gaze shift latency (uncued overlap) ‡ | 0.04 | 0 | 0.62 | 45 | >.90 | -0.29 |
| Gaze shift latency (uncued gap) | -0.01 | 0 | -0.12 | 47 | >.90 | -0.01 |
| Gaze shift variability (uncued) | -0.1 | 0 | -0.66 | 47 | >.90 | -0.68 |
| Pupil-dilation response (cued overlap) | -0.5 | -3.61 | -2.7 | 46 | **.048*** | 0.07 |
|  |  |  |  |  |  |  |
| **Relation to EXT change** |  |  |  |  |  |  |
| Alerting effect | 0.01 | 0 | 0.25 | 43 | >.90 | 0.22 |
| Gaze shift latency (uncued overlap) ‡ | -0.058 | 0 | -0.61 | 43 | >.90 | 0.55 |
| Gaze shift latency (uncued gap) | 0.01 | 0 | 0.06 | 45 | >.90 | 0.07 |
| Gaze shift variability (uncued) | 0.06 | 0 | 0.27 | 46 | >.90 | -21.07 |
| Pupil-dilation response (cued overlap) | -0.95 | -6.8 | -3.6 | 44 | **.004**** | 0.76 |

* *p* < .05 (Bonferroni-corrected). EXT = externalizing symptoms. ‡ 1 outlier observation removed. Unstandardized *b* coefficients indicate the predicted change in ms (gaze shift latency) or pupil size (mm). ΔAIC = difference in Aikaike information criterion (AIC) between GLMs fitted with identity versus log link.

*Table S4. Relations between eye movement measures, internalizing symptoms (INT) at T2, and change in INT symptoms from T1 to T2. (General linear models fit with* ***log link*)**

|  | **b** | **β** | | **t** | **df** | **P (Bonferroni)** | **ΔAIC (identity – log link)** |
| --- | --- | --- | --- | --- | --- | --- | --- |
| **Relation to INT symptoms at T2** |  |  |  |  |  |  |  |
| Alerting effect | -0.03 | 0 | | -1.43 | 45 | .799 | 0.25 |
| Gaze shift reaction time (silent overlap) | -0.01 | 0 | | -0.18 | 45 | >.90 | -0.26 |
| Gaze shift reaction time (silent gap) | -0.09 | 0 | | -1.58 | 47 | .602 | 0.23 |
| Gaze shift variability (uncued) | 0.02 | 0 | | 0.15 | 47 | >.90 | -0.81 |
| Pupil-dilation response | 0.07 | 0.47 | | 0.44 | 46 | >.90 | 0.06 |
|  |  |  | |  |  |  |  |
| **Relation to INT change** |  |  | |  |  |  |  |
| Alerting effect | -0.06 | 0 | | -2.48 | 43 | .086 | 0,51 |
| Gaze shift latency(silent overlap) | -0.09 | 0 | | -1.16 | 43 | >.90 | 0,04 |
| Gaze shift latency(gap) | 0 | 0 | | 0.04 | 45 | >.90 | 0,53 |
| Gaze shift variability (uncued) | 0.07 | 0 | | 0.37 | 45 | >.90 | -0,55 |
| Pupil-dilation response (cued overlap) | -0.06 | -0.41 | | -0.29 | 44 | >.90 | 0,31 |

* *p* < .05 (Bonferroni-corrected). INT = internalizing symptoms. ‡ 1 outlier observation removed. Unstandardized *b* coefficients indicate the predicted change in MS (gaze shift latency) or pupil size (mm). ΔAIC = difference in Aikaike information criterion (AIC) between GLMs fitted with identity versus log link.

S5. Change in experimental measures during the course of the task

Exploratory analyses were conducted to examine change in the experimental measures over the course of the task. For each condition, data were arranged into blocks of three trials. Linear mixed effects models (LMME) were fitted with main and interaction effects of ADHD symptoms at T1 and (blocked) trial for each condition. Random intercept for participant and random slopes for trial were included to account for repeated measures. Statistical significance was tested with an analysis of variance (ANOVA) with Satterthwaite corrected degrees of freedom.

To visualize the results, participants were split into three groups based on their level of ADHD symptoms at T1 (high symptom group: > 66^th^ percentile; low symptom group: <33^th^ percentile, and mid symptom group: 33-66^th^ percentile).

*Gaze shift reaction time.*

As can be seen in *Table S2,* no significant main or interaction effects involving trial or ADHD symptoms were found. However, as expected, a main effect of condition was found, driven by a difference between the gap and overlap conditions. *Figure S7* shows mean gaze shift reaction time as a function of trial and condition in participants split in three groups according to ADHD symptom level (high: > %75, low: <%25, mid, %25-%75)

*Table S5. Results from linear mixed effects model examining gaze shift reaction time as a function of trial, ADHD symptoms at T1, and condition.*

| **Effect** | **NumDF** | **DenDF** | **F** | **P** |
| --- | --- | --- | --- | --- |
| ADHD | 1 | 202.83 | 2.65 | .105 |
| trial | 1 | 723.33 | 0.41 | .523 |
| condition | 2 | 698.87 | 15.24 | **<.001***** |
| ADHD * trial | 1 | 735.63 | 0.00 | .988 |
| ADHD * condition | 2 | 700.28 | 0.07 | .931 |
| trial * condition | 2 | 701.00 | 2.24 | .107 |
| ADHD * trial * condition | 2 | 704.65 | 1.07 | .344 |

*NumDF = Numerator degrees of freedom; DenDF = Satterthwaite approximated denominator degrees of freedom. *** p <.001*

*
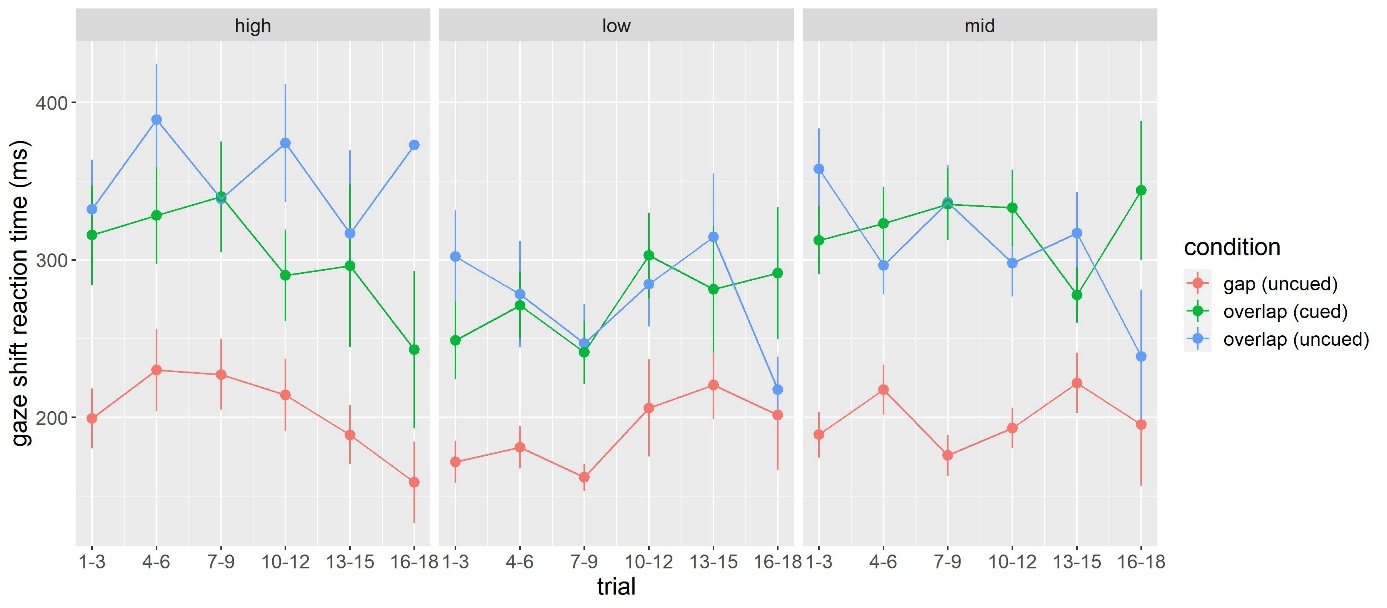
*

*Figure S7. Mean gaze shift reaction time as a function of trial and condition in participants split according to symptom level* (high: > %75, low: <%25, mid, %25-%75). *Error bars show standard errors.*

*Pupil dilation response*

*As can be seen in Figure S8,* a reliable pupil dilation response was observed in the cued overlap condition indicated by values above zero, but not to the uncued overlap or gap conditions.

Significant main effects of trial and condition as well as a trial x condition interaction effect were found (all *p <.*001, see *Table S3 and Figure S8*). No main or interaction effects with ADHD were found, indicating that change in pupil dilation response over the course of the experiment did not interact with ADHD symptoms (see *Table S3*). Follow-up tests of the trial x condition interaction using Kenward-Roger estimated degrees of freedom showed that pupil dilation responses decreased over time in the cued overlap condition (b = -0.029, t = -7.016, p <.001) whereas no effect of trial was found in the uncued overlap (b = -0.007, t = -1.639, p = .091) or the gap conditions (b = 0.006, t = 1.478, p = .140). Since reliable pupil dilation responses were only found in the cued overlap condition, we repeated the analysis in this condition separately. Again, no interaction effect between trial and ADHD symptoms was found (F (2,38.46) = 0.46, p = .500).

*Table S6. Results from linear mixed effects model examining pupil dilation as a function of trial, ADHD symptoms at T1, and condition.*

| **Effect** | **NumDF** | **DenDF** | **F** | **P** |
| --- | --- | --- | --- | --- |
| ADHD | 1 | 332,11 | 1,52 | .219 |
| trial | 1 | 734,27 | 11,06 | **<.001***** |
| condition | 2 | 699,94 | 92,10 | **<.001***** |
| ADHD * trial | 1 | 746,43 | 0,11 | .742 |
| ADHD * condition | 2 | 702,47 | 2,06 | .129 |
| trial * condition | 2 | 703,99 | 11,50 | **<.001***** |
| ADHD * trial * condition | 2 | 709,88 | 0,10 | .902 |

*NumDF = Numerator degrees of freedom; DenDF = Satterthwaite approximated denominator degrees of freedom. *** p <.001*

*
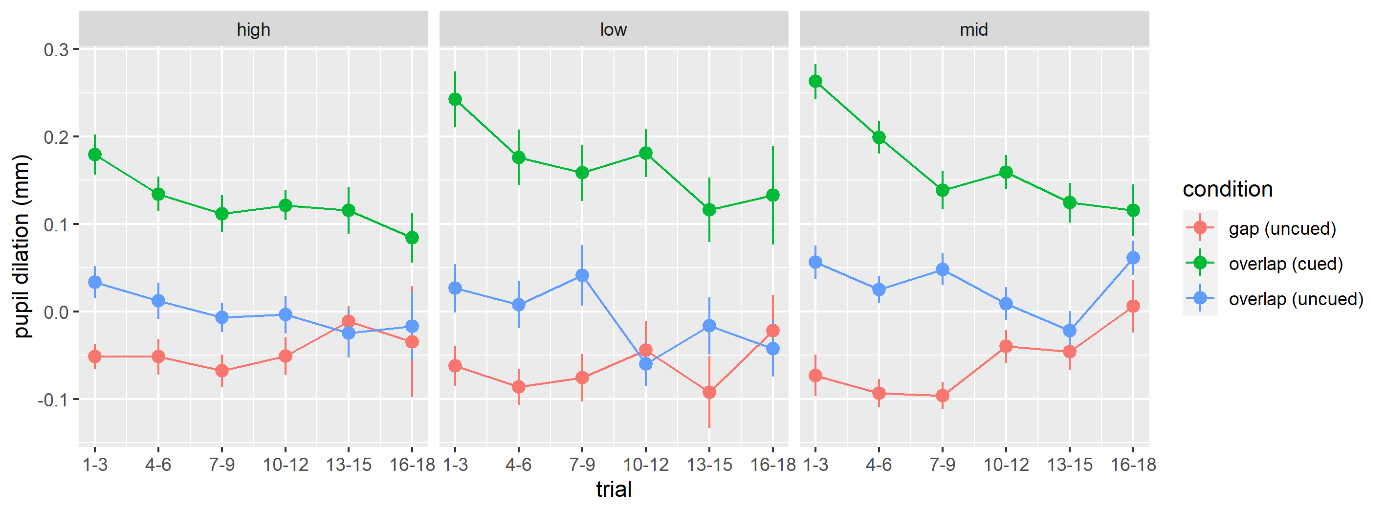
*

*Figure S8. Mean pupil dilation (mm) as a function of trial and condition in participants split according to symptom level* (high: > %75, low: <%25, mid, %25-%75). *Error bars show standard errors.*

S6. Relation between symptom measures and gaze shift reaction times in the cued overlap condition

*Table S7. Results from general linear models examining gaze shift reaction times in the cued overlap condition as a function of ADHD, Internalizing, and Externalizing symptoms*

| **Analysis** | **b** | **β** | **t** | **df** | **P (Bonferroni)** |
| --- | --- | --- | --- | --- | --- |
| Relation to ADHD at T2 | 0.28 | 0 | 0.02 | 48 | >.90 |
| Relation to ADHD change | 0.36 | 0 | 0.01 | 47 | >.90 |
| Relation to EXT at T2 | -3.21 | -0.02 | -0.12 | 47 | >.90 |
| Relation to EXT change | -18.66 | -0.14 | -0.47 | 45 | >.90 |
| Relation to INT at T2 | 15.58 | 0.11 | 0.66 | 47 | >.90 |
| Relation to INT change | 35.06 | 0.26 | 1.09 | 45 | >.90 |

*NumDF = Numerator degrees of freedom; DenDF = Satterthwaite approximated denominator degrees of freedom. *** p <.001. INT = Internalizing symptoms; EXT = Externalinzing symptoms*
